# Research Progress in the Synthesis of Carbon Dots and Their Application in Food Analysis

**DOI:** 10.3390/bios12121158

**Published:** 2022-12-12

**Authors:** Yuan Yu, Lili Zhang, Xin Gao, Yuanmiao Feng, Hongyuan Wang, Caihong Lei, Yanhong Yan, Shuiping Liu

**Affiliations:** 1 Zhejiang Provincial Key Laboratory of Fiber Materials and Manufacturing Technology, Zhejiang Sci-Tech University, Hangzhou 310018, China; 2 Zhejiang Provincial Innovation Center of Advanced Textile Technology, Shaoxing 312000, China; 3 College of Textile and Clothing, Yancheng Institute of Technology, Yancheng 224051, China

**Keywords:** carbon dots, optical properties, food analysis, application

## Abstract

Food safety is connected to public health, making it crucial to protecting people’s health. Food analysis and detection can assure food quality and effectively reduce the entry of harmful foods into the market. Carbon dots (CDs) are an excellent choice for food analysis and detection attributable to their advantages of good optical properties, water solubility, high chemical stability, easy functionalization, excellent bleaching resistance, low toxicity, and good biocompatibility. This paper focuses on the optical properties, synthesis methods, and applications of CDs in food analysis and detection, including the recent advances in food nutritional composition analysis and food quality detection, such as food additives, heavy metal ions, foodborne pathogens, harmful organic pollutants, and pH value. Moreover, this review also discusses the potentially toxic effects, current challenges, and prospects of CDs in basic research and applications. We hope that this review can provide valuable information to lay a foundation for subsequent research on CDs and promote the exploration of CDs-based sensing for future food detection.

## 1. Introduction

With the continuous progress and development of technology, the variety of food products is becoming more and more diverse. At the same time, food safety issues [1] are emerging. On the one hand, the variety and complexity of food production makes food testing and detection a challenge. On the other hand, to seek higher profits, some harmful additives, such as heavy metal ions, pesticides, and antibiotics, are left in the food and greatly affect human health. To prevent emerging food safety problems, there is an urgent requirement to strengthen food testing [2]. Therefore, it is of utmost concern to develop simple, fast, and accurate analytical methods for the analysis of food ingredients and the rapid detection of harmful elements.

In recent years, with the increasing emphasis on food safety, rapid food testing techniques have developed rapidly. Compared with surface-enhanced Raman spectroscopy (SERS), high-performance liquid chromatography (HPLC), and enzyme-linked immunosorbent assays (ELISA), which are commonly used in traditional analyses and testing techniques [3,4,5,6,7,8], carbon dots (CDs) probe detection has the advantages of simple preparation process, speed, high sensitivity, low cost, and low requirements for instruments and equipment. The CDs probes have broad application prospects in the field of food testing, which is gaining the interest of many researchers.

CDs are quasi-spherical nanoparticles with a size of less than 10 nm, and their basic constituent elements include C, H, and O. To optimize the optical properties of CDs, elements such as N, S, P, B, F, Mn are doped into the CDs [9]. The abundant groups on the surface of CDs endow them with many excellent physicochemical properties. For many CDs, heteroatomic doping is an effective method to improve the properties of CDs. Shrestha et al. [10] synthesized CDs by using phosphoric acid (H_3_PO_4_) as an activating agent so that the CDs were successfully applied as an adsorbent material for the removal of rhodamine B dye. Protr et al. [11] studied the effect of melamine addition on CDs properties.

Xu et al. [12] discovered a new technique of CDs serendipitously in 2004. Sun et al. [13] obtained fluorescent carbon nanoparticles by laser etching in 2006, which have excellent fluorescence properties in both solution and solid states, resistance to photobleaching, and no scintillation effect. Compared with organic dyes, semiconductors, and metal quantum dots, CDs have good biocompatibility, low toxicity, water solubility, tunable luminescence range, and photostability [14,15,16]. The toxicity of CDs has been investigated according to the previous research. The toxicity of CDs in vitro could be confirmed by 3-(4,5-dimethylthiazol-2-yl)-2,5-diphenyltetrazolium bromide (MTT) assays. Zhang et al. [17] reported that CDs have nearly no toxicity in high concentrations in Hela cells. Sahu et al. [18] reported that CDs were nontoxic to L929 cells in the high concentration of 200 μg/mL. The toxicity of CDs in vivo was explored by many researchers. Yang et al. [19] found CDs had no toxicity on mice even at high dosages. Therefore, CDs are the ideal candidate for food analysis and detection.

The developments and applications of CDs are expanding into biological, optoelectronic, fluorescent anti-counterfeiting, and fluorescent detection. Depending on the composition of the carbonaceous core, there are three major categories: graphene quantum dots, carbon nanodots, and polymer dots. Graphene quantum dots refer to a class of fully carbonized CDs with a single-layer or multi-layer structure of graphite, while carbon nanodots and polymer dots can be classified as products of incomplete carbonization of CDs during the synthesis process. The difference between the two is that carbon nanodots are mostly prepared from precursors such as small molecules and retain uncarbonated functional groups on the surface, while polymer dots are mostly formed with the involvement of polymers, and there are some polymer chains on the surface or there is an obvious cross-linked polymeric structure.

The morphology and elemental composition of CDs are generally characterized by atomic force microscopy (AFM), transmission electron microscopy (TEM), X-ray photoelectron spectroscopy (XPS), X-ray diffractometry (XRD), and Fourier transform infrared spectroscopy (FTIR). TEM and AFM can visualize the morphology and size of CDs, typically 1–5 nm in height and 1–10 nm in diameter. XRD, XPS, and FTIR can provide the properties of the lattice structure, elemental composition, content, and surface functional groups of CDs [20,21,22,23].

## 2. Preparation of CDs

Since the discovery of CDs, there has been continuous research for more efficient synthetic paths to further improve the optical properties of CDs and expand their applications, which are mainly divided into two techniques: top-down and bottom-up [24,25,26,27].

### 2.1. Top-Down Technique

**Figure 1:**
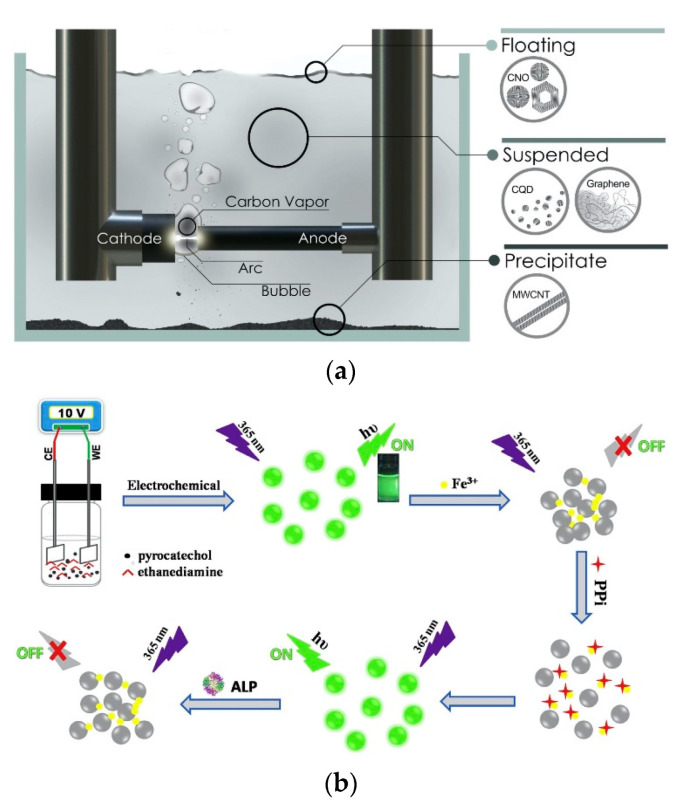
(**a**) Schematic illustration of CDs synthesis by arc discharge. Reprinted with the permission from Ref. [29]. Copyright 2022, AIP Publishing. (**b**) Schematic illustration of CDs synthesis and the detection strategies for PPI and ALP activity based on the aggregation and disaggregation of the CDs. Reprinted with the permission from Ref. [38]. Copyright 2022, Elsevier.

The top-down technique refers to the physical or chemical decomposition of large carbon skeletons, such as graphite rods, carbon nanotubes, carbon fibers, candle ash, carbon black, and other large particle size carbon-based materials, to form CDs with relatively small dimensions. The main methods include laser ablation, arc discharge, acidic oxidation, and electrochemical synthesis. The top-down process always requires complex reaction conditions and expensive equipment. Moreover, the preparation process generates a large amount of waste liquid, which is not conducive to large-scale production and practical application. Laser ablation is a kind of method based on high temperature and high pressure and the energy ablation of carbon source generated by a laser to obtain CDs. It is a useful technique for producing CDs with a finite size distribution, high water solubility, and fluorescence. Sun et al. [13] first used laser irradiation of carbon powder and binder and after a multi-step process of oxidation and passivation, CDs were eventually obtained with improved fluorescence properties. Cui et al. [28] fabricate CDs and applied them in biomedical imaging by the dual-beam laser ablation method. Chao et al. [29] synthesize CDs with a quantum yield (QY) of 16%, and the wavelengths were 275 nm (A band) and 285 nm (B band) by arc discharge, as shown in Figure 1a. The CDs obtained by arc discharge have good water solubility but contain many impurities that are difficult to extract and purify. The acidic oxidation method generally uses strong oxidizing reagents to oxidize and corrode carbon sources such as coal tar, candle ash, and coke to obtain CDs [30,31]. Feng’s group [32] produced CDs from coke by a simple and green chemical oxidation method for a new application. The prepared CDs emitted blue fluorescence, and the results showed that the fluorescence QY was 9.2%. They have also been found to have superior application potential in lighting devices. Iannazzo [33] used acid oxidation technology to oxidize multi-walled carbon nanotubes (MWNTs) for a long time, exfoliated them to prepare water-soluble GQDs, and studied the antiviral activity of graphene-based nanomaterials for targeting HIV therapy, drug therapy, drug delivery, etc. The electrochemical synthesis method is to use a simple electrochemical device to apply a certain voltage to the working electrode, and under the action of electrolysis, the precursor substances are oxidized, aggregated, carbonized, and passivated, thereby preparing CDs [34,35,36]. Sun et al. [37] synthesized FeN-CDs with QY of 7.5% by an electrochemical method. Niu et al. [38] prepared nitrogen-doped CDs with green fluorescence using catechol and ethylenediamine as raw materials with a QY of 30.6%, as shown in Figure 1b.

### 2.2. Bottom-Up Method

The bottom-up method uses small organic molecules or oligomers as the carbon source to prepare CDs through a series of processes such as dehydration, polycondensation, and carbonization. The main methods are hydrothermal/solvent, microwave-assisted, template/carrier, ultrasound, and combustion. Common sources of carbon are polyethylene glycol [39], urea [40], citric acid [41], glucose [42], and natural materials such as garlic [43], lotus leaf [44], coffee [16], banana peel [45], etc. The bottom-up method has low equipment requirements, simple operation, and high QY and is currently a widely reported method for the synthesis of CDs.

**Figure 2:**
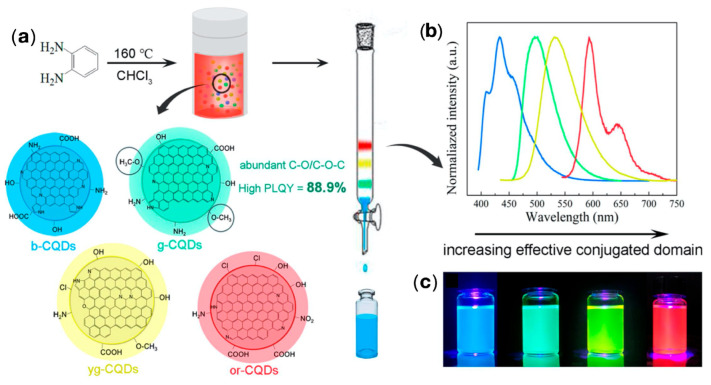
(**a**) One-pot synthesis and purification for full-color CDs, (**b**) normalized PL spectra under optimal excitations (the colors of the lines correspond to the fluorescent color of the CDs), and (**c**) photographs under a 395 nm UV lamp. Reprinted with the permission from Ref. [48]. Copyright 2021, ACS.

**Figure 3:**
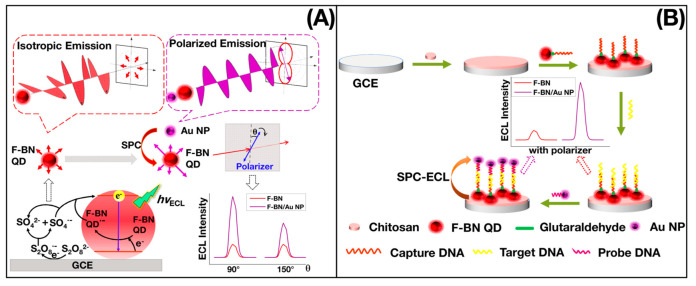
(**a**) Polarized SPC-ECL mechanism of CDs; (**b**) schematic illustration of polarized ECL sensor. Reprinted with the permission from Ref. [54]. Copyright 2020, ACS.

The hydrothermal/solvothermal technique synthesizes relatively insoluble materials under conventional conditions by using a reaction kettle under high temperature in the presence of an aqueous solvent or mineralizer, and a heterogeneous chemical reaction takes place to dissolve and recrystallize [46,47]. Zhang et al. [48] hydrothermally synthesized blue, green, yellow–green and orange–red CDs by a one-pot hydrothermal method using o-phenylenediamine as a carbon source, with QY of 88.9%, as shown in Figure 2. He et al. [49] synthesized water-soluble CDs with bright blue–green emission under UV or blue light irradiation, showing a homogeneous morphology and good crystallinity. The microwave-assisted method is an effective method for the synthesis of CDs, which is obtained by heating and carbonizing the carbon source using the radiation energy of electromagnetic waves [50,51,52,53]. Liang et al. [54] synthesized F-doped CDs from boric acid, C_3_H_6_N_6_, and NH_4_F by the microwave-assisted hydrothermal method. This kind of CDs possessed unique photoluminescent blue and electrochromic yellow properties, as shown in Figure 3. The ultrasonic method synthesizes CDs by ultrasonically gathering heat from carbon sources in a short time with a high-power ultrasound instrument. In 2011, Li et al. [55] first synthesized CDs by a one-step ultrasonic method using activated carbon as a carbon source. Subsequently, Li et al. [56] reported the use of glucose under acidic or alkaline conditions to obtain water-soluble CDs, which were fluorescently upconverted and stabilized at room temperature for 6 months. The combustion method is a method with a simple process, energy saving, and high purity. Generally, wood, paper, n-butanol, and candles are incompletely burned to obtain CDs. However, due to incomplete combustion, it does not conform to the concept of full utilization and environmental friendliness. Zhang et al. [57] used ethanol, household candles, n-butanol, and benzene as ingredients to make CDs by the combustion method, revealing the structure and photoluminescence properties of CDs. Cheng et al. [58] efficiently synthesized high-yield N/S co-doped CDs (N/S-CDs) based on cellulose-based willow wool biowaste in a simple one-step combustion process. The resulting N/S-CDs showed an increased yield of 14.3% and an average diameter of 7.3 nm, exhibiting excellent photostability, low cytotoxicity, and pH stability. Miao et al. [59] reported the synthesis of citric acid and urea into multi-color-emitting CDs by controlled graphitization and surface functionality. The multi-color emitting CDs were simply prepared for large-scale synthesis, with high QY and good photoluminescence intensity.

### 2.3. Fluorescent Properties of CDs

CDs attract many researchers’ attention largely due to their unique fluorescence properties. The fluorescence emission wavelength of CDs has a wide range, which can extend from the visible light area to the near-infrared area, making up for the shortage of traditional organic reagents in the near-infrared area. There is no definitive conclusion regarding the fluorescence mechanism of CDs. The core of CDs is a carbon nanoparticle dominated by sp^2^-hybridized C atoms with abundant functional groups on the surface. Two commonly accepted views on the fluorescence mechanism of CDs are intrinsic state/carbon-core fluorescence and surface/edge-state fluorescence. Dong et al. [40] modified the CDs by introducing other elements (oxygen, nitrogen, and sulfur) into the carbon skeleton and explored the influence of doping with other elements on the fluorescence properties of CDs. The results showed that oxygen-doped CDs had a complex surface state that produced a range of different energy levels and therefore possessed a broader absorption and emission spectrum. After nitrogen doping, a new energy level is created on the original complex surface state, and the electrons are bound by the emerging energy level, thus producing a fluorescence enhancement effect. So far, the mechanism of surface state fluorescence has not been fully determined, but it can be inferred that fluorescence groups and energy traps on the surface are likely to be the mechanism of surface state fluorescence.

Most CDs have excitation-dependent emissions; that is, the emission wavelength is significantly red-shifted with increasing excitation wavelengths. However, the causes of this phenomenon are not clear and the structure of the CDs, such as defect states, functional groups, and size, may affect the excitation light dependence of the CDs. Zheng et al. [60] reported the excitation light-related properties may be related to surface defects in aromatic C=C and groups such as C-OH and C=O. Li et al. [61] postulated that the surface defects and narrower size distribution of CDs were probably related to their excitation light-related properties. Researchers also reported excitation-dependent emissions may be attributed to the uniform size and surface state of the CDs [62]. The CDs also possess pH-sensitive properties, which may be the result of protonation and deprotonation of functional groups on the surface of CDs. In recent years, fluorescent pH sensor-based CDs were extensively prepared for applications.

Upconversion fluorescence properties are generally found during the sonication of synthetic CDs. Ma et al. [63] synthesized water-soluble fluorescent N-doped CDs (NCDs) in a one-pot sonication reaction with glucose and ammonium hydroxide, which exhibited strong fluorescence in the visible to near-infrared range. The obtained NCDs also exhibited upconversion fluorescence properties in the range of 300–600 nm with excitation times in the range of 650–1000 nm. Owing to the high penetration of long wavelength excitation light (such as the near infrared region NIR), CDs are used for molecular imaging with high spatial resolution and low background interference, which has its unique advantages in the fields of biological sample detection and imaging.

The chemiluminescence (CL) of CDs is attributed to the compounding of positively charged CDs and negatively charged CDs produced in the system to form excited CDs, which produce chemiluminescence upon return to the ground state. In 2011, Lin et al. [64] first discovered that H_2_O_2_ can directly oxidize CDs for fluorescence, which is very similar to some semiconductor quantum dots [65,66]. Chen et al. [67] prepared S, N co-doped CDs with ultra-high QY (79%) by hydrothermal method, and the N, S-CDs enhanced the chemiluminescence intensity of the luminol-H_2_O_2_ system. Ranitidine could quench the chemiluminescence intensity of this system with a linear range of 0.5–50 g mL^−1^ and a detection limit of 0.12 g ml^−1^.

Electrochemiluminescence (ECL) is an important optical property of CDs. ECL sensors based on CDs and graphene dots have been showing great potential and achievements in recent decades. Zheng’s group [68] prepared CDs by electrochemical oxidation of graphite, which had good ECL properties, suggesting that CDs may have surface defects. The study showed that the CDs have the advantages of strong and stable ECL emission, excellent water solubility, good stability, low cytotoxicity, easy labeling, and environmental friendliness, which have promising applications in the development of biosensors in the future. The introduction of CDs into the field of ECL provides new ideas and strategies for the development of new reagents to design ECL sensors.

## 3. Application of CDs in Food Testing

CDs have excellent properties such as high fluorescence intensity, excellent chemical stability, resistance to photobleaching, and good biocompatibility. CDs possess significant advantages in fluorescence sensing analysis and are often applied as fluorescence sensing probes with a very broad application prospect in food analysis [69]. Food analysis and detection is an essential measure to ensure food safety by analyzing the nutritional composition of food and the presence of toxic and harmful ingredients in food. The harmful ingredients in the food are mainly pathogenic bacteria, heavy metal ions, pesticide residues, and chemicals, excessive intake of which can cause great harm to human health. Moreover, pH detection is also important in measuring the quality of food. Changes in pH often accompany food spoilage, so it is necessary to monitor the quality of food during production, processing, storage, and distribution to maintain food safety.

### 3.1. Nutritional Composition Analysis of Food

Food nutrient analysis provides a reference for the daily nutrition and energy intake of humans, such as proteins, vitamins, fats, and minerals, which are necessary for the normal life activities of the human body. The rapid development of CDs has been applied to sensing probes for the detection and analysis of various nutrients in food because of their good optical properties and high sensitivity. The fluorescence quenching caused by the surface electron transfer between CDs and metal ions is often used in the analysis of food nutrients.

Glutathione (GSH), as a redox buffer in the cells of living organisms, is an important antioxidant and free radical scavenger in the body. Xu et al. [70] invented a fluorescence sensor for the rapid, sensitive and selective detection of GSH in food samples by hydrothermal synthesis of CDs. The binding of mercury to CDs leads to the turning off CDs’ fluorescence, and in the presence of GSH could turn on the fluorescence of the sensor. This sensor had been successfully applied to the determination of GSH in a variety of food samples, covering a wide range and with an LOD of 37 nmol/L. Based on this study, researchers replace mercury with copper, thus reducing toxicity and achieving effective detection of GSH in food [71].

Ovalbumin (OVA) is an essential source of protein in food and its levels are used as a reference for evaluating protein quality. Fu et al. [72] established a fast and universal CDs-based sensor for the detection of OVA. The sensor had a linear response range of 0.5 to 15 μg/mL to OVA, which was applied to the quantitative detection of OVA in egg products.

**Figure 4:**
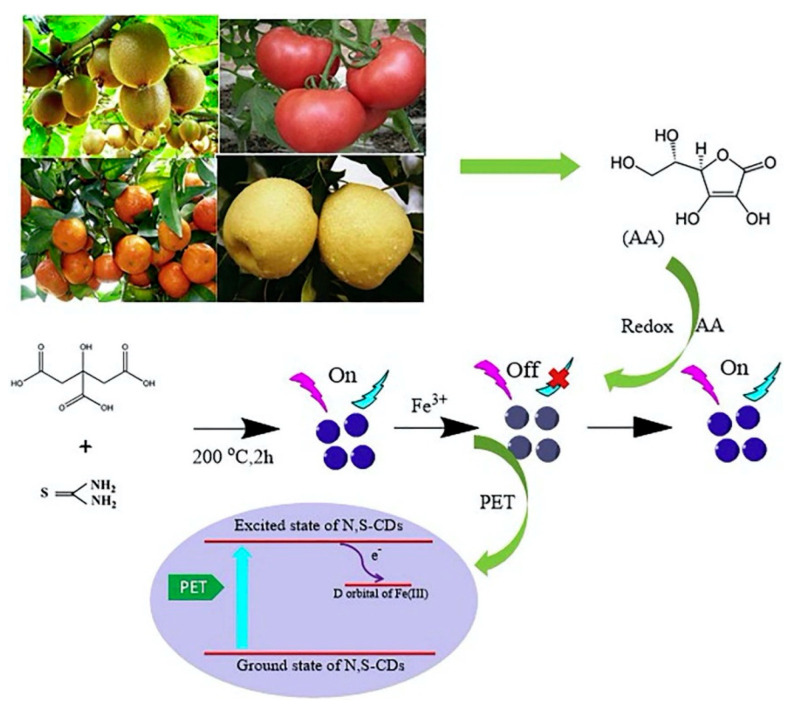
Schematic illustration of the N, S-CDs/Fe^3+^ system for detection of AA. Reprinted with the permission from Ref. [74]. Copyright 2022, Elsevier.

Vitamin C (VC), which is also known as L-ascorbic acid, is a water-soluble vitamin that is widely found in various vegetables and fruits [73]. Excessive VC added to food increases the risk of urinary stones, skin cancer, and other diseases, so it is necessary to develop an effective sensor to test the content of VC in food. Luo et al. [74] developed an N and S co-doped CD (N, S-CD) as an “off-on” fluorescence sensor to VC with a detection range of 10–200 μmol/L and LOD of 4.69 μmol/L, as shown in Figure 4. The high selectivity and stability of the constructed sensor were successfully used for the determination of vitamin C in common fruits.

### 3.2. Food Additive Detection

Food additives play an essential role in prolonging the shelf life of food, enhancing the flavor of food and improving food quality. However, the excessive use of legal additives (including colorants and preservatives) and the addition of prohibited additives (including melamine, Sudan red, and others) in food additives can pose a huge threat to human health. The linearity between the fluorescence intensity and the concentration of the analyte is fundamental for the quantitative detection of a wide range of food additives. The selectivity and sensitivity of CDs in food additive detection are greatly enhanced after appropriate modifications.

**Figure 5:**
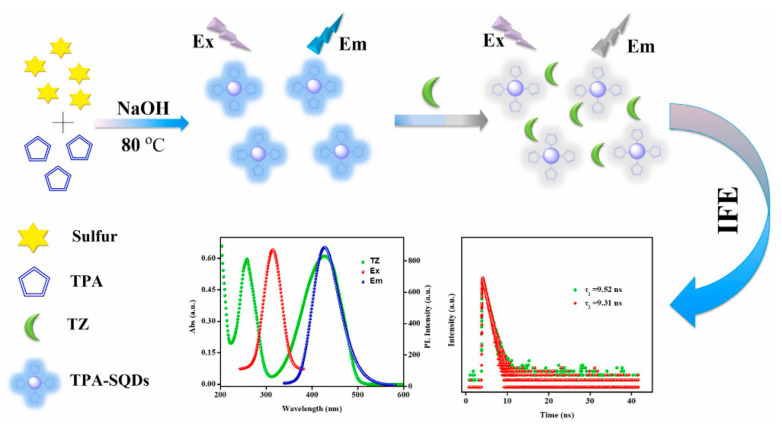
Preparation strategy of fluorescent TPA-SQDS and schematic diagram for TZ detection. Reprinted with the permission from Ref. [76]. Copyright 2022, Elsevier.

**Figure 6:**
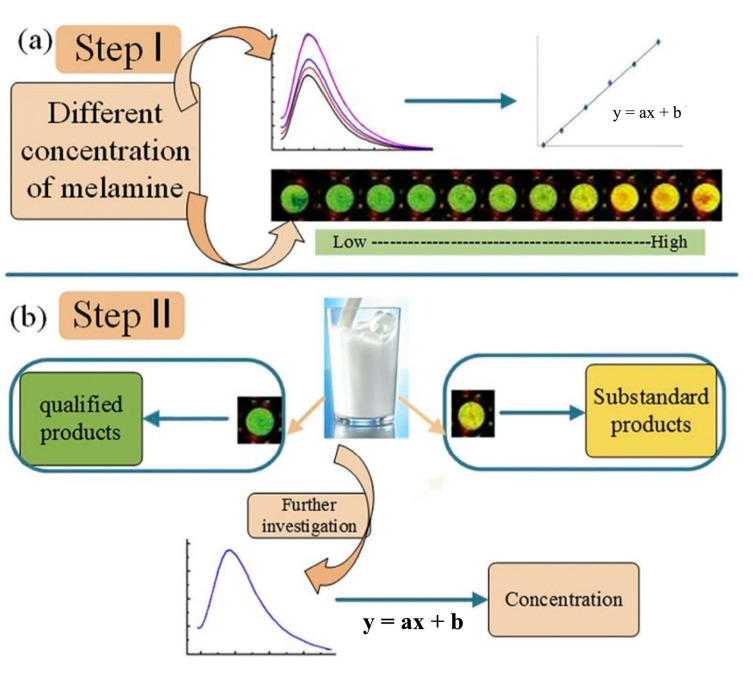
(**a**) Step I: development of a Au@CQDs-based fluorescence method for melamine detection. (**b**) Step II: detection of milk adulterated by melamine. Reprinted with the permission from Ref. [79]. Copyright 2018, Elsevier.

Lemon yellow is a synthetic coloring agent used to improve the appearance of food. Previous studies have shown that excessive intake of artificial colors can affect children’s mental development and even pose serious health risks. Li et al. [75] synthesized sulfur-doped CDs (S-CDs) from hollow rosemary and thiourea. The S-CQDs possessed excellent fluorescence properties, with a QY of 20.19%, and a selective reaction with lemon yellow, resulting in a fluorescence-quenching effect with a detection limit of 0.45 μmol/L. Similarly, Peng et al. [76] synthesized S-CDs protected by terephthalic acid as a stabilizer with a QY of 85.99% and LOD of 39 nmol/L, which could be applied to detect citric yellow in beverages, as shown in Figure 5. Xu et al. [77] used 3,4,9,10-tetracarboxylic dianhydride (PTD) as a carbon source to synthesize CDs that could be used to detect sunset yellow FCF. Sunset yellow FCF selectively quenched the fluorescence of CDs by fluorescence resonance energy transfer (FRET). The CDs showed excellent selectivity and sensitivity for FCF in the detection of food additives. Lin et al. [78] prepared serine-modified CDs using glycerol as the carbon source and PEG1500 as the passivating agent by a microwave-assisted method. Hu et al. [79] designed a Au@CDs nanocomposite that showed enhanced fluorescence emission with increasing melamine concentration. The approximate concentration of melamine adulterated in milk samples could be detected visually by a smartphone, as shown in Figure 6. The detection limit of the sensor was 3.6 nM. Yang et al. [80] prepared sulfur-doped fluorescent CDs with excellent stability in acidic environments with a hydrothermal method of lignin in sulfuric acid solution. These CDs possessed good stability with a pH range of 0–5.0, and were successfully applied as a sensor for the detection of Sudan I in acidic solutions with a linear range of 0–40 μM and a detection limit of 0.12 μM.

### 3.3. Detection of Foodborne Pathogens

Food safety accidents caused by foodborne diseases have occurred repeatedly around the world. Common pathogens causing foodborne diseases include Salmonella typhimurium, E-coli, helicobacter pylori, vibrio parahaemolyticus, etc. [81,82]. Weng et al. [83] prepared fluorescent CDs from solid ammonium citrate and mannose with a one-step dry-heat method with a QY of 9.8%. Based on the ability of mannose to specifically bind to *E. coli* lectins, the labeling of *E. coli* was achieved, thus allowing a simple and rapid method for the quantitative detection of *E. coli* at low concentration levels (103 cfu/mL) in various samples such as tap water and fruit juices. Helicobacter pylori is a pathogenic microorganism of gastric cancer, which is usually transmitted through contaminated water and unclean food. Chattopadhyay et al. [84] prepared a fluorescent CDs probe for the sensitive detection of H.pylori based on a fluorescence resonance energy transfer (FRET). The linear detection range was 5–107 cells/mL and LOD was 10 cells/mL.

Salmonella food poisoning can lead to headache, dizziness, nausea and life-threatening dehydration and acidotic heart failure in severe cases. Wang et al. [85] prepared a novel fluorescent probe that attached amino-modified aptamers to the surface of carboxy-modified CDs to form an aptamer. The CDs aptamer complex (CD-apt) for sensitive quantitative detection of Salmonella typhi had a detection range of 10^3^–10^5^ cfu/mL and a detection limit as low as 50 cfu/mL. Long-term consumption of foods containing low concentrations of aflatoxin (AFM1) is the main cause of some cancer diseases. Singh et al. [86] synthesized blue fluorescent N-doped CDs with citric acid and polyethyleneimine by hydrothermal method. The CDs/Ab probes were obtained by immobilizing anti-AFM1 antibodies (Ab) on N-doped CDs. The fluorescence of the CDs/Ab was effectively quenched with increasing concentrations of AFM1, with a sensitivity in the range of 0.2–0.8 ng/mL and a detection limit of 0.07 ng/mL.

### 3.4. Detection of Metal Ions

Heavy metals such as silver, mercury, and copper are all non-biodegradable and accumulate in the human body, which can cause various health problems. The sensitive quantification of heavy metal ions is of considerable importance because of their potential effects on humans. Currently, nanoprobes based on CDs have been designed to achieve the quantitative detection of heavy metal ions such as Cu^2+^, Hg^2+^, Al^3+^, Ag^+^, Pb^2+^, and Mn^2+^ [87,88,89].

Liu et al. [90] prepared CDs with Chinese grass carp scales (CGCS) by an environmentally friendly method. Hg^2+^ had a strong affinity and unique selectivity with the prepared CGCS-CDs. CGCS-CDs could be used as specific fluorescent probes for the detection of Hg^2+^ ions in the concentration range of 0.014–30 μmol/L with a LOD of 0.014 μmol/L. Wei et al. [91] prepared red fluorescent CDs (R-CDs) using sodium citrate and formamide in a one-step solvent method with a fluorescence QY of 35.3%. Cu^2+^ could selectively quench the fluorescence intensity of R-CDs, with a detection limit as low as 5 nmol/L. Qin et al. [92] prepared S/N-CDs for the effective detection of Ag^+^. The S/N-CDs were synthesized by a hydrothermal method with citric acid and guanidine thiocyanate by co-doping with N and S. The fluorescence intensity of S/N-CDs was quenched in the presence of Ag^+^ and was not interfered with by other metal ions, indicating that the CDs had good selectivity for Ag^+^.

### 3.5. Residue Detection of Hazardous Organic Pollutants

To meet the food needs of the growing world population, the use of pesticides and veterinary drugs has become more intensive. However, it is inevitable that some organic contaminants such as pesticides, veterinary drugs, and antibiotics will remain in the food, including prototypes of drugs and their metabolites that accumulate in food, all of which are potentially hazardous to human health [93,94,95,96]. Emerging CDs can provide more accurate, efficient and cost-effective fluorescent sensing probes, and in recent years, they have made great progress in the detection of hazardous organic contaminant residues.

To develop a simple and economical method for the detection of organophosphorus pesticides in vegetable samples. Yan et al. [97] synthesized CDs from 3-aminophenylboronic acid by one-step hydrothermal method. The fluorescence of CDs could be quenched by MnO_2_ nanosheets based on the FRET mechanism, but in the presence of butyrylcholinesterase (BChE) and acetylthiocholine, the enzymatic product (thiocholine) decomposes the MnO_2_ nanosheets, leading to the recovery of the fluorescence of the CDs. Therefore, an “on/off” fluorescence sensing system was developed in the range of 0.05–5 ng/mL with a detection limit of 0.015 ng/mL.

**Figure 7:**
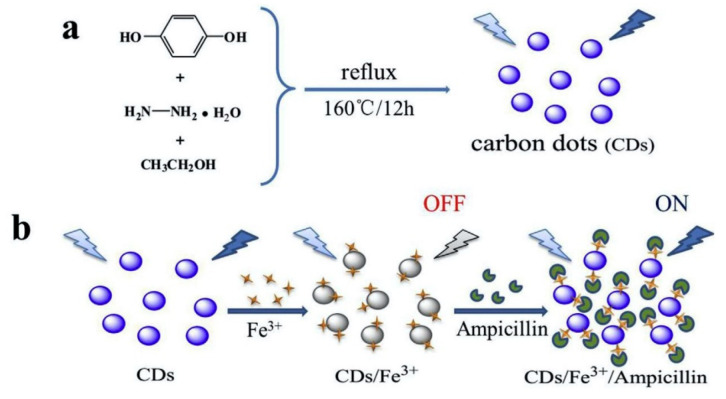
Schematic diagrams showing (**a**) the synthesis of CDs and (**b**) the sensing mechanisms of CDs for Fe^3+^and ampicillin. Reprinted with the permission from Ref. [99]. Copyright 2022, Elsevier.

In addition to the common organophosphorus pesticide residues, there are other harmful small-molecule residues in food products. Zhang et al. [98] synthesized a sulfur-doped CDs containing a molecularly imprinted polymer (MIP) and sensitized with ionic liquid, which could be used for the detection of pesticide residues in vegetables and tea by photosensitive λ-cyhalothrin with a linear range of 1–150 μg/kg, a detection limit of 0.5 μg/kg, and a recovery of 98.90–116.93%. Fu et al. [99] synthesized CDs emitting blue fluorescence from dihydroxybenzene and hydrazine hydrate. The fluorescence of CDs was able to be quenched by strong metal-ligand coordination and electrostatic interactions with Fe^3+^, as shown in Figure 7. Further studies revealed that the complex exhibited fluorescence turn-on to ampicillin with a detection limit of 0.70 μM, which could be used for the detection of ampicillin in mineral water, milk, and pork samples.

### 3.6. pH Detection

The detection of food pH values can not only judge the maturity of fruits and vegetables and the freshness of food but also reflect the quality indicators of food. In addition, acid plays a significant role in maintaining the acid–base balance of human body fluids. Therefore, obtaining accurate pH values quickly can not only provide a reference for food sterilization, storage, microbial fermentation, etc. but also help the human body to control the intake of acidic food, which is conducive to human health.

Few methods are currently available for pH determination using CDs as sensors. Ma et al. [100] prepared CDs based on the Maillard reaction (MR-CDs) using L-tryptophan and D-glucose, which exhibited stable pH-dependent behavior and showed an excellent optical linear response to pH in the range of 4.0–7.5 and 7.5–13.0. Zhu et al. [101] prepared fluorescent CDs by a hydrothermal method using natural kelp as a carbon source and modified the surface of the CDs with polyethyleneimine (PEI), which was conjugated with fluorescein isothiocyanate (FITC) to synthesize a CDs-FITC composite. The CDs-FITC composite was prepared as a sensor for the dual-signal fluorescence detection of pH and Cu^2+^ ions. Luo et al. [102] prepared a biological CDs by a one-step hydrothermal method, showing excellent reversibility and photostability in pH measurements, and the photoluminescence intensity was linearly related to the pH values from 6 to 12 in buffer solutions.

## 4. Conclusions and Prospects

CDs, as a new type of optical nanosensing material, possess the advantages of excellent fluorescence, low toxicity, good biocompatibility, easy preparation, low cost, anti-photobleaching, good chemical stability, etc. and have a broad application prospect in the field of food detection [103]. However, the development of CDs for food analysis is still at the preliminary stage of research. Although researchers prepared functionalized CDs with the ability to target the identification of heavy metals, pesticides, veterinary drug residues, food additives, and foodborne pathogens in food, there are still many issues that need to be addressed. Therefore, targeted doping and modification of CDs is a core component in the design of CDs sensors.

The fluorescence mechanism of CDs is still unclear because of the variety of carbon sources and the different methods of preparation. Compared to semiconductor quantum dots, the fluorescence QY of CDs is relatively low and their internal structure and functional group distribution are uncertain. The preparation of CDs is still at the laboratory stage, with poor repeatability and stability, and the subsequent purification is often complex and time-consuming. Moreover, according to the characteristics of the CDs structure, most functionalized CDs have a wide intrinsic energy band gap and a short fluorescence emission wavelength. CDs can only be used as fluorescent energy acceptors or light source signal donors, which makes CDs sensors suffer from greater interference during detection. The complexity of the food matrix in real sample detection limits the specificity and sensitivity of CDs-based assays to a certain extent. Therefore, the expansion of multifunctional detection of CDs is extremely meaningful. At the same time, by selecting appropriate carbon sources and optimizing the preparation process, improving the fluorescence QY, reusability, and chemical stability of CDs will also make great contributions to the industrialization of CDs. In addition, doped or surface-functionalized CDs combined with immunoassays, molecularly imprinted polymer MIPs, and electrochemical sensors are gradually emerging to improve the sensitivity, accuracy, and specificity of detection, which is important to further broaden the application of CDs.

With food safety issues becoming increasingly serious, the development of fast and functional detection devices is an important means of achieving a full-chain-coverage food traceability detection system. Compared to traditional detection methods, CDs have greater advantages in terms of preparation costs, handling methods, safety of use, and chemical stability. It is believed that soon, fast and multi-functional CDs fluorescent sensors for food safety will be designed and widely used. In the future, we have the following expectations: It is necessary to further prepare the CDs by more economical and simple methods. The green and nature raw synthesis materials should be further explored to reduce the burden on the environment. In order to improve the stability and detection limit of CDs, the surface modification technology needs to be further explored.

## Data Availability

Not applicable.
